# Unravelling the pharmacokinetics of aflatoxin B1: *In vitro* determination of Michaelis–Menten constants, intrinsic clearance and the metabolic contribution of CYP1A2 and CYP3A4 in pooled human liver microsomes

**DOI:** 10.3389/fmicb.2022.988083

**Published:** 2022-08-29

**Authors:** Orphélie Lootens, Marthe De Boevre, Elke Gasthuys, Jan Van Bocxlaer, An Vermeulen, Sarah De Saeger

**Affiliations:** ^1^Department of Bioanalysis, Centre of Excellence in Mycotoxicology and Public Health, Ghent University, Ghent, Belgium; ^2^Department of Bioanalysis, Laboratory of Medical Biochemistry and Clinical Analysis, Ghent University, Ghent, Belgium; ^3^MYTOX-SOUTH^®^, International Thematic Network, Ghent, Belgium; ^4^Department of Biotechnology and Food Technology, University of Johannesburg, Johannesburg, Gauteng, South Africa

**Keywords:** mycotoxins, CYP450 enzymes, pharmacokinetic, *in vitro*, Michaelis–Menten constant, LC–MS/MS, aflatoxin B1, human liver microsomes

## Abstract

Mycotoxins, fungal secondary metabolites, are ubiquitously present in food commodities. Acute exposure to high levels or chronic exposure to low levels has an impact on the human body. The phase I metabolism in the human liver, performed by cytochrome P450 (CYP450) enzymes, is accountable for more than 80% of the overall metabolism of exogenous and endogenous compounds. Mycotoxins are (partially) metabolized by CYP450 enzymes. In this study, *in vitro* research was performed on CYP450 probes and aflatoxin B1 (AFB1), a carcinogenic mycotoxin, to obtain pharmacokinetic data on AFB1, required for further experimental work. The CYP450 probes of choice were a CYP3A4 substrate, midazolam (MDZ) and a CYP1A2 substrate, phenacetin (PH) since these are the main metabolizing phase I enzymes of AFB1. Linearity experiments were performed on the three substrates indicating that linear conditions were achieved at a microsomal protein concentration and incubation time of 0.25 mg/ml and 5 min, 0.50 mg/ml and 20 min and 0.25 mg/ml and 5 min for MDZ, PH and AFB1, respectively. The *K_m_* was determined in human liver microsomes and was estimated at 2.15 μM for MDZ, 40.0 μM for PH and 40.9 μM for AFB1. The associated *V*_max_ values were 956 pmol/(mg.min) (MDZ), 856 pmol/(mg.min) (PH) and 11,536 pmol/(mg.min) (AFB1). Recombinant CYP systems were used to determine CYP450-specific Michaelis–Menten values for AFB1, leading to a CYP3A4 *K_m_* of 49.6 μM and an intersystem extrapolation factor (ISEF) corrected *V*_max_ of 43.6 pmol/min/pmol P450 and a CYP1A2 *K_m_* of 58.2 μM and an ISEF corrected *V*_max_ of 283 pmol/min/pmol P450. An activity adjustment factor (AAF) was calculated to account for differences between microsome batches and was used as a correction factor in the determination of the human *in vivo* hepatic clearance for MDZ, PH and AFB1. The hepatic blood clearance corrected for the AAF CL_H,B,MDZ,AAF_, CL_H,B,PH,AAF_ CL_H,B,AFB1,AAF(CYP3A4)_ and CL_H,B,AFB1,AAF(CYP1A2)_ were determined in HLM at 44.1 L/h, 21.7 L/h, 40.0 L/h and 38.5 L/h. Finally, inhibition assays in HLM showed that 45% of the AFB1 metabolism was performed by CYP3A4/3A5 enzymes and 49% by CYP1A2 enzymes.

## Introduction

Mycotoxins are secondary metabolites produced by fungi. Mycotoxin contamination is a global food safety issue leading to major public health concerns. Mycotoxins are observed on food crops such as maize, wheat, sorghum and peanuts (Eskola et al., 2019). They are produced by fungi as a self-protection mechanism during stressful conditions, and can be toxic to humans and animals, causing illness, or even death (Bennett and Klich, 2003; Kamala et al., 2018; Pleadin et al., 2019). Fungi are able to produce multiple mycotoxins, which leads to the co-existence of a great number of metabolites leading to agonistic or even synergistic effects, causing (co)-morbidities and pathologies (Sobral et al., 2018). Aflatoxin B1 (AFB1) is a human carcinogenic mycotoxin produced by *Aspergillus flavus* and *Aspergillus parasiticus*. Cancer, hepatotoxicity and immunosuppression are linked to AFB1 (IARC, 2002; Rotimi et al., 2019; Claeys et al., 2020). The exposure to these food contaminants is often chronic and substantial, depending on the region of the world. High levels of contamination occur in regions where no strict regulations for mycotoxins are applied or where awareness is lacking, e.g., on the African continent (Kebede et al., 2020). Repeated exposure to multiple mycotoxins not only has an impact on public health in general, but could more specifically also lead to interactions with other xenobiotic substances – such as medicinal drugs – in the body by altering their pharmacokinetics (PK) and/or pharmacodynamics (PD). In order to get insight in these food contaminants and their interactions with medicinal drugs, knowledge of their PK is required.

Metabolism occurs in different parts of the human body, though mainly by hepatic enzymes, and is divided in phase I (oxidation, hydrolysis and reduction), predominantly through the action of cytochrome P450 (CYP450) enzymes (Zhou et al., 2009), and phase II (conjugation) reactions (Jancova et al., 2010). Considering all enzymes, the CYP450 complex is an important group involved in phase I metabolism, mainly found in liver and gut. It is the most relevant enzyme family to consider in view of its predominant role in drug metabolism since it is involved in approximately 80% of all drug metabolism processes, as well as in the metabolism of endogenic compounds (Hannemann et al., 2007). Aflatoxins (AFs) are metabolized via CYP450 to different metabolites such as aflatoxin M1 (AFM1), aflatoxin Q1 (AFQ1), aflatoxin-exo-8,9-epoxide and aflatoxin-endo-8,9-epoxide (AFBO) (Ivanova et al., 2019). CYP1A2, CYP3A4, CYP2A13 and CYP3A5 are involved in AFB1’s metabolism (Gallagher et al., 1996; Bbosa et al., 2013). Having insight into ‘what the body does to the compound’ is important to have an idea on potential interactions and is also necessary to understand what effects the compound will have in the body. Different types of metabolic interactions are possible, potentially leading to higher or lower effects of the co-administered drugs. The measurement of enzyme activities and the determination of how the metabolic rate changes in the presence of enzyme inhibitors or inducers is important in drug development, additionally to detect possible interactions. Insight in these parameters is not only crucial for drug compounds but also for other compounds such as food contaminants. Drug–drug interactions are imperative when bringing new compounds to the market, but it is also crucial to check for interactions with other substances such as food-contaminants to which one is exposed on a daily basis. In addition, variability in expression and activity of CYP450 enzymes is known to occur in humans and animals, mostly due to genetic polymorphisms (Tracy et al., 2016). But also, factors such as age, gender, ethnicity and health status can have an impact on CYP450 enzyme expression and activity (Yang et al., 2010). Clearly, covering these interplaying effects requires in-depth starting knowledge of *in vitro* data.

This is the first detailed report of PK parameters of AFB1 studied via *in vitro* research in human liver microsomes (HLMs) and recombinant systems (rhCYPs) using liquid chromatography tandem mass spectrometry (LC–MS/MS). Furthermore, the PK parameters of CYP450 probe substrates were determined to avoid interlaboratory differences and to confirm the applied *in vitro* methods by comparing our results to literature data.

## Materials and methods

### Linearity experiments and *K_m_*, *V*_max_ determination

#### Chemicals and reagents

Dipotassium hydrogen phosphate, potassium dihydrogen phosphate, and KCl were purchased from VWR (Oud-Heverlee, Belgium). Acetonitrile (ACN) was purchased from Biosolve B.V. (Valkenswaard, The Netherlands), and formic acid (FA) from Merck (Darmstadt, Germany). Trifuoroacetic acid (TFA) was purchased from Fluka Chemicals (Buchs, Switzerland). Ethyl acetate was purchased from Acros (New Jersey, United States). Water was from a Ultrapure water system (Sartorius, Goettingen, Germany). LC–MS grade methanol and glacial acetic acid were purchased from Biosolve B.V. (Valkenswaard, The Netherlands). Ammonium acetate was from Merck (Darmstadt, Germany). Midazolam (MDZ) was purchased from Roche (Mannheim, Germany). Phenacetin (PH) and chlorpropamide (CHL) were purchased from Sigma Aldrich (St. Louis, MO, United States). Nicotinamide adenine dinucleotide phosphate tetrasodium (NADPH.4Na) was purchased from Gentaur (Kampenhout, Belgium) and stored at −20°C. The stock solutions of the probe substrates, the metabolites and the internal standard were prepared separately in methanol (MeOH) at a concentration of 1 mg/ml and stored at −20°C. HLMs (Corning^®^ UltraPool™ HLM 150, Mixed Gender, 0.5 ml), for experimental use, were purchased from Corning (Woburn, United States), information on the preparation of the HLM can be found in the product description sheet. The HLM were stored at −80°C. AFB1 and zearalanone (ZAN) were purchased from Fermentek (Jerusalem, Israel). Supersomes human CYP3A4 + oxidoreductase (1,000 pmoL/mL) were purchased from Corning (Woburn, United States) and human CYP1A2 low reductase (LR) EasyCYP bactosomes (1,000 pmoL/mL) were purchased from Tebu-Bio (Boechout, Belgium) and stored at −80°C. Ketoconazole (CYP3A4 inhibitor) and α-naphthoflavone (CYP1A2 inhibitor) were purchased from Sigma Aldrich (St. Louis, MO, United States) and stored at 4°C. All chemicals and reagents were of analytical grade. SimCYP (Certara^©^) was used for the prediction of certain PK parameters.

#### Preparation of standard and work solutions (linearity experiments)

Stock solutions of MDZ, PH, AFB1, ZAN and CHL at a concentration of 1 mg/ml were prepared in MeOH and stored at −20°C (AFB1 at 4°C). Work solutions of MDZ, PH and AFB1 were made in Ultrapure water at a concentration of 5 μM, 20 μM and 5 μM, respectively. MDZ was used as a CYP3A4 probe substrate, PH as a CYP1A2 probe substrate to include the two main metabolizing CYP450 enzymes in the metabolism of AFB1 (Gallagher et al., 1996; Bbosa et al., 2013). Phosphate buffer of 0.2 M and pH of 7.4 was made and frozen at −20°C in 60 ml tubes. NADPH.4Na was freshly made every experimental day at a concentration of 5 mM in phosphate buffer (pH 7.4). A 1.15% KCl solution was made in HPLC grade water and stored at 4°C. HLM (20 mg/ml in 250 mM sucrose) were diluted in 1.15% KCl to achieve a final protein concentration of 0.1; 0.25; 0.5; 0.75 and 1 mg/ml in the samples. The stop reagent was made with 300 μl FA, 5,500 μl ACN and Ultrapure water and contained the internal standards (IS; 0.072 μM of CHL and 0.12 μg/ml of ZAN).

#### Microsomal incubation and sample clean-up (linearity experiments)

Samples were prepared partially based on Schelstraete et al. (2019a,b). Centrifugal Eppendorf cups were filled with 50 μl substrate solution (i.e., 5 μM for MDZ and AFB1 and 20 μM for PH), 50 μl 1.15% KCl and 50 μl 0.2 M phosphate buffer. Next, 50 μl of a freshly prepared NADPH.4Na solution was added. After an incubation of 3 min, 50 μl of diluted HLM was added and they were placed back on the thermoshaker TS-100 (Biosan, Geraardsbergen, Belgium) with a rotation speed of 300 rpm and at a temperature of 37°C. After the indicated time (i.e., 5, 10, 20, 30 and 45 min), 25 μl of an ice cold stop reagent with IS, i.e., CHL for CYP probes and ZAN for AFB1, was added. For protein precipitation, 125 μl of TFA was added. Samples were centrifuged at 16,000*×g* for 20 min at 4°C. The centrifugal filters were discarded and the supernatant was added to 1 ml of a 0.2 M phosphate buffer. Next, 7 ml of ethyl acetate was added in order to perform a liquid–liquid extraction (LLE). Samples were extracted during 20 min on an Agitelec shaker at room temperature. For phase separation, samples were centrifuged at 2,000*× g* for 10 min. Subsequently, the organic phase was transferred in glass tubes and evaporated under a gentle nitrogen stream at 40°C (±5°C) using a Turbovap (Biotage, Charlotte, United States). After evaporation, samples were kept at −20°C until further analysis. Upon analysis, 250 μl of a 60/40 mobile phase A (MP A; H_2_O/MeOH/acetic acid (94/5/1, v/v/v) + 5 mM ammonium acetate)/mobile phase B (MP B; H_2_O/MeOH/acetic acid (2/97/1, v/v/v) + 5 mM ammonium acetate) mixture was added. After thorough vortexing and centrifuging for 10 min at 1,000*× g*, the samples were transferred to an autosampler vial. An aliquot of 5 μl was injected into the ultrahigh performance liquid chromatography (UPLC) - XEVO TQ-S (LC–MS/MS) equipment using an *in-house* developed and validated method.

#### LC–MS/MS (linearity experiments)

A Waters Acquity class I UPLC system coupled to a XEVO TQ-S tandem quadrupole mass spectrometer (MS) from Waters (Milford, MA, United States) was used for the detection and quantification. A Charged Surface Hybrid (CSH) C_18_ column (1.7 μm 2.1 × 100 mm) with Guard column was used for chromatographic separation. The column temperature was set at 30°C; the sample temperature at 10°C. The MP was used at a flow rate of 0.250 ml/min following a gradient program (Table 1). The total duration for a single run was 12 min. The MS was operated in the electrospray positive mode (ESI+) with multiple reaction monitoring (MRM). The MRM parameters for the CYP probes and for AFB1, respectively, are given in Table 2. Source temperature and desolvation temperature were set at 130°C and 200°C. The cone gas flow was set at 150 L/h, the desolvation gas flow at 550 L/h. Data was processed using Masslynx^®^ and Targetlynx^®^ software from Micromass (Manchester, United Kingdom). Data analysis was performed by transferring Targetlynx^®^ data to Microsoft Excel and Sigmaplot version 14.5. For AFB1, substrate depletion was measured. Nonlinear regression on sigmoidal plots where the initial depletion rate (k_dep_) was plotted against AFB1 concentrations on a linear-log plot, was performed using Excel Solver (Youdim and Dodia, 2010).

**Table 1:** Gradient program used for the liquid chromatography–tandem mass spectrometry (LC–MS/MS) at a constant flow rate of 0.25 ml/min.

Time (min)	Solvent A (%)	Solvent B (%)	Curve
Initial	60	40	Initial
2.5	60	40	1
3.5	55	45	6
8.5	10	90	7
10	60	40	6
12	60	40	1

The numbers in the last column are linked to a shape of a curve that represents the gradient of the mobile phases in that specific time interval. Solvent A = H_2_O/MeOH/acetic acid (94/5/1, v/v/v). Solvent B = MeOH/H_2_O/acetic acid (97/2/1, v/v/v).

**Table 2:** Multiple reaction monitoring transitions and mass spectrometry settings for the CYP probes, aflatoxin B1 (AFB1) and internal standards used.

Channel	Retention time (min)	Precursor ion (Da)	Product ion (Da)	Dwell (s)	Cone (V)	Collision (eV)
Midazolam						
1	5.16	326.0	223.0	0.033	50.00	30.00
2	5.16	326.0	249.0	0.033	50.00	30.00
3	5.16	326.0	291.0	0.033	50.00	20.00
α-OH-Midazolam						
1	6.95	342.0	203.0	0.033	25.00	22.00
2	6.95	342.0	289.0	0.033	25.00	22.00
3	6.95	342.0	324.0	0.033	25.00	20.00
Phenacetin						
1	3.32	180.1	110.0	0.026	40.00	13.00
2	3.32	180.1	138.0	0.026	40.00	10.00
3	3.32	180.1	152.0	0.026	40.00	10.00
Acetaminophen						
1	1.23	152.1	93.00	0.108	40.00	20.00
2	1.23	152.1	110.0	0.108	40.00	10.00
3	1.23	152.1	134.0	0.108	40.00	8.00
Chlorpropamide (IS)						
1	6.33	277.0	111.0	0.033	30.00	25.00
2	6.33	277.0	175.0	0.033	30.00	12.00
3	6.33	277.0	192.0	0.033	30.00	7.00
Aflatoxin B1						
1	5.07	313.0	241.1	0.033	65.00	32.00
2	5.07	313.0	270.1	0.033	70.00	35.00
Zearalanon (IS)						
1	9.13	321.0	189.1	0.033	35.00	22.00
2	9.13	321.0	303.3	0.033	35.00	14.00

IS, internal standard.

#### Preparation of standard and work solutions *K_m_* – *V*_max_-experiments in HLM and rhCYPs

The same stock solutions of MDZ, PH, CHL, AFB1 and ZAN were used at a concentration of 1 mg/ml in MeOH and stored at −20°C (apart from AFB1, which was stored at 4°C). Probe work solutions of MDZ were made in Ultrapure water to achieve sample concentrations in a range from 0.1 μM to 100 μM. For PH, sample concentrations ranged from 1 μM to 200 μM. Substrate work solutions of AFB1 were made to achieve sample concentrations from 0.5 μM to 50 μM, for the HLM experiments. For rhCYP an extra AFB1 sample concentration of 100 μM was implemented. HLM (20 mg/ml in 250 mM sucrose) were diluted in 1.15% KCl to achieve a final protein concentration of 0.25 and 0.5 mg/ml in the samples, as based on previous linearity experiments. The stop reagents were those described in 2.1.2.

#### Sample preparation and clean-up *K_m_* – *V*_max_-experiments in HLM and rhCYPs

Samples were treated as described in Section “Microsomal incubation and sample clean-up (linearity experiments),” but the HLM protein concentrations were 0.25 mg/ml for MDZ and 0.5 mg/ml for PH and AFB1. For CYP3A4 rhCYP experiments, 50 μl of diluted supersomes at 150 pmol/ml were used instead of HLM, achieving a final concentration of 30 pmol/ml. For CYP1A2 rhCYP experiments, 50 μl of diluted EasyCYP LR bactosomes at 50 pmol/ml were used, achieving a final concentration of 10 pmol/ml. The incubation time was 5 min for MDZ and AFB1 and 20 min for PH.

#### LC–MS/MS *K_m_* – *V*_max_-experiments in HLM and rhCYPs

The same equipment, settings and procedures were used as explained in Section “LC–MS/MS (linearity experiments).” Data analysis was performed by transferring Targetlynx^®^ data to Microsoft^®^ Excel. Metabolite formation velocity versus substrate concentration figures were plotted with error bars, Sigmaplot version 14.5 was used for determining the Michaelis–Menten constant (*K_m_*) and maximum velocity (*V*_max_) by performing nonlinear regression on the figures. For AFB1 substrate depletion was measured. Nonlinear regression on sigmoidal plots, where the initial depletion rate (k_dep_) was plotted against AFB1 concentrations on a linear-log plot, was performed using Excel Solver (Youdim and Dodia, 2010).

#### Applied formulas

The obtained *K_m_* and *V*_max_ values of the CYP probes and AFB1 from HLM are used to calculate the intrinsic *in vitro* clearance (CL_int,_*_in vitro_*; Equation 1) and the *in vivo* intrinsic hepatic clearance rate (CL_H,int_; Equation 2) with the use of a microsomal protein per gram liver (MPPGL) of 40 mg/g and a liver weight (LW) of 1,650 g (Kazmi, 2015).


(1) $${\mathrm{CL}}_{\mathrm{int},in\;vitro}\left(\frac{\mu L}{\mathrm{mg}\times \min}\right)=\frac{V_{\max}\left(\frac{\mu \mathrm{mol}}{\min\times \mathrm{mg}\mathrm{protein}}\right)}{K_{m}\left(\frac{\mu \mathrm{mol}}{L}\right)*10^{-6}\frac{L}{\mu L}}$$

(2) $${\mathrm{CL}}_{H,\mathrm{int}}\left(\frac{L}{h}\right)={\mathrm{CL}}_{\mathrm{int},in\;vitro}*\mathrm{MPPGL}*\mathrm{LW}*\frac{60\min}{h}*10^{-6}\frac{L}{\mu l}$$

The free microsomal fraction (f_u,mic_) for AFB1 was estimated with the use of Equation 3 for neutral compounds.


(3) $$f_{u,\mathrm{mic}}=\frac{1}{\left[\left(\mathrm{protein concentration}\;\left(\frac{\mathrm{mg}}{\mathrm{mL}}\right)*10^{0.522*\mathrm{logP}-1.728}\right)+1\right]}$$

The unbound *in vivo* intrinsic hepatic clearance rate (CL_H,u,int_) is calculated by dividing the CL_H,int_ by the free microsomal fraction (f_u,mic_) as shown by Equation 4.


(4) $${\mathrm{CL}}_{H,u,\mathrm{int}}\left(\frac{L}{h}\right)=\frac{{\mathrm{CL}}_{H,\mathrm{int}}}{f_{u,\mathrm{mic}}}$$

Next, the activity adjustment factor (AAF; T’jollyn et al., 2017) was calculated using the PK values of the probe substrates MDZ in the case of CYP3A4 and PH in the case of CYP1A2. For the estimation of the f_u,mic_ Equation 5 was used where f_u,2_ equals f_u,mic_. The AAF (Equation 6) is obtained by dividing unbound *in vivo* CL_int_ for a specific CYP450 enzyme (CL_int,u,*in vivo*,CYP_) by the unbound HLM CL_int_ for a specific CYP450 enzyme (CL_int,u,HLM,_CYP).


(5) $$f_{u,2}={\left[\left(\frac{C2}{C1}*\frac{\left(1-f_{u,1}\right)}{f_{u,1}}\right)+1\right]}^{-1}$$

(6) $$\mathrm{AAF}=\frac{{\mathrm{CL}}_{\mathrm{int},u,invivo,\mathrm{CYP}}}{{\mathrm{CL}}_{\mathrm{int},u,\mathrm{HLM},\mathrm{CYP}}}$$

The CL_H,u,int_ is multiplied by the AAF, leading to an AAF corrected CL_H,u,int_ (CL_H,u,int,AAF_)_._ The obtained *K_m_* and *V*_max_ values of the CYP probes from rhCYPs were used to calculate intersystem extrapolation factors (ISEFs) for both *V*_max_ and CL_int_ (Equations 7 and 8).


(7) $$V_{\max}\;\mathrm{ISEF}=\frac{V_{\max,\mathrm{HLM}}}{V_{\max,\mathrm{rhCYP}}*\mathrm{CYP}\;\mathrm{abundance}\;\left(\mathrm{pmol}\;P450/\mathrm{mg}\right)}$$

(8) $${\mathrm{CL}}_{\mathrm{int}}\;\mathrm{ISEF}=\frac{\frac{V_{\max,\mathrm{HLM}}}{\left(K_{m,\mathrm{HLM}}*10^{-6}\right)}}{\frac{V_{\max,\mathrm{rhCYP}}}{\left(K_{m,\mathrm{rhCYP}}*10^{-6}\right)}*\mathrm{CYP}\;\mathrm{abundance}\;\left(\mathrm{pmol}\;P450/\mathrm{mg}\right)}$$

CL_H,u,int,AAF_ values of AFB1 from rhCYP experiments were multiplied with the CL_int_ ISEF, resulting in an ISEF corrected CL_H,u,int,AAF_ (CL_H,u,int,AAF,ISEF_). The extrapolation to the whole liver hepatic blood clearance from HLM experiments is shown in Equation 9, assuming a human hepatic blood flow of 90 L/h (Q_H_). Equation 10 presents the extrapolation to the whole liver hepatic blood clearance from rhCYP experiments. The hepatic blood clearance (CL_H,B_) was multiplied by the blood/plasma ratio (B:P) to obtain the whole liver hepatic plasma clearance, CL_H,P._


(9) $${\mathrm{CL}}_{H,B}\left(\frac{L}{h}\right)=\frac{Q_{H}*f_{u,b}*{\mathrm{CL}}_{H,u,\mathrm{int},\mathrm{AAF}}}{Q_{H}+f_{u,b}*{\mathrm{CL}}_{H,u,\mathrm{int},\mathrm{AAF}}}$$

(10) $${\mathrm{CL}}_{H,B}\left(\frac{L}{h}\right)=\frac{Q_{H}*f_{u,b}*{\mathrm{CL}}_{H,u,\mathrm{int},\mathrm{AAF},\mathrm{ISEF}}}{Q_{H}+f_{u,b}*{\mathrm{CL}}_{H,u,\mathrm{int},\mathrm{AAF},\mathrm{ISEF}}}$$

### Inhibition assay AFB1

#### Preparation of standard and work solutions inhibition assay AFB1

A 1 mg/ml solution in MeOH was prepared of ketoconazole, a selective CYP3A4 inhibitor, and α-naphthoflavone, a selective CYP1A2 inhibitor, used in inhibition assays as mentioned in guidelines from the European Medicines Agency and the Food and Drug Administration (Sai et al., 2000; EMA, 2012; Food and Drug Administration, 2020).

The same stock solutions of AFB1 and ZAN were used at a concentration of 1 mg/ml in MeOH (previously described) and stored at −20°C.

A substrate solution of AFB1 was made in Ultrapure water to obtain a final sample concentration of 5 μM. The ketoconazole stock solution was diluted with MeOH leading to a 50 μM solution. The α-naphthoflavone stock solution was diluted with MeOH to a 250 μM solution. Phosphate buffer of 0.2 M and a pH of 7.4 was made and frozen at −20°C in 60 ml tubes. NADPH.4Na was freshly prepared every experimental day at a concentration of 5 mM in phosphate buffer (pH 7.4). A 1.15% KCl solution was made in Ultrapure water and stored at 4°C. The HLM (20 mg/ml in 250 mM sucrose) were diluted in 1.15% KCl to achieve a final protein concentration of 0.5 mg/ml in the samples. The stop reagent is the same as described in Section “Chemicals and reagents.”

#### Sample preparation, clean-up and LC–MS/MS inhibition assays AFB1

Samples were prepared based on an *in-house* validated method (Schelstraete et al., 2019a,b). Briefly, centrifugal Eppendorf cups were filled with 50 μl of 5 mm NADPH.4Na, 50 μl 1.15% KCl and 50 μl 0.2 M phosphate buffer and put on a thermoshaker TS-100 (Biosan, Geraardsbergen, Belgium) with a rotation speed of 300 rpm and a temperature of 37°C. Next, 50 μl of a 2.5 mg/ml HLM suspension was added leading to a final concentration of 0.5 mg/ml in the sample. Next, 5 μl of an inhibitor in methanol was added, i.e., 50 μM of ketoconazole (CYP3A4 inhibitor) leading to a final concentration of 1 μM in the sample and 250 μM of α-naphthoflavone (CYP1A2 inhibitor) leading to a final concentration of 5 μM. The inhibitors were added in small volumes to minimize solvent effects (2%). After adding the inhibitor, the samples were equilibrated for 5 min. AFB1 was added in a volume of 50 μl and a concentration of 25 μM leading to a sample concentration of 5 μM. After incubating for 10 min, 25 μl of stop reagent with IS was added. Control samples were ran without inhibitors at a protein concentration of 0.5 mg/ml and incubation time of 10 minutes to be able to determine the inhibited fraction. Next, the cups were vortexed and put on ice. They were then stored at −20°C. The next day, a sample clean-up was performed. The sample clean up and sample preparation are similar as described previously in 2.1.3. An aliquot of 5 μl was injected into the LC–MS/MS using an *in-house* developed and validated method. The same equipment, settings and procedures were used as depicted in section “LC–MS/MS (linearity experiments).” Data analysis was performed by transferring Targetlynx data to Microsoft Excel.

## Results

### Determination of optimal incubation conditions, *K_m_* and *V*_max_ for CYP probes and AFB1

**Figure 1:**
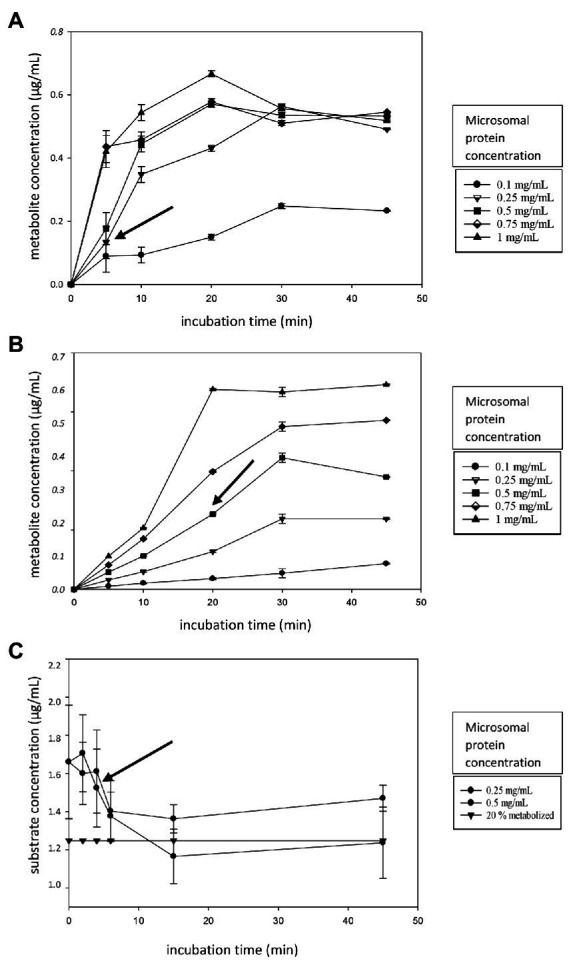
**(A–C)** Linearity experiment figures. **(A)** The metabolite formation of alpha-hydroxy-midazolam (α-OH-MDZ, **A**) and acetaminophen **(B)** over time (0–45 min) for different microsomal protein concentrations (0.10–1.0 mg/ml). **(C)** The AFB1 depletion over time (0–45 min) for different microsomal protein concentrations (0.25–0.50 mg/ml). The horizontal curve (black triangles) represents the 20% metabolite formation threshold. Incubations were performed in triplicate, error bars are displayed. The black arrow indicates the chosen microsomal protein concentration and incubation time.

Linearity experiments were performed in HLM to obtain data on the ideal incubation time and microsomal protein concentration for further determination of the *K_m_* and *V*_max_. The obtained results are shown in Figures 1A–C. A microsomal protein concentration of 0.25 mg/ml and an incubation time of 5 min were chosen for MDZ (Figure 1A, black arrow). For PH, the protein concentration was 0.5 mg/ml with a 20 min incubation time (Figure 1B, black arrow), while for AFB1, a microsomal protein concentration of 0.5 mg/ml and an incubation time of 5 min were chosen (Figure 1C, black arrow).

**Figure 2:**
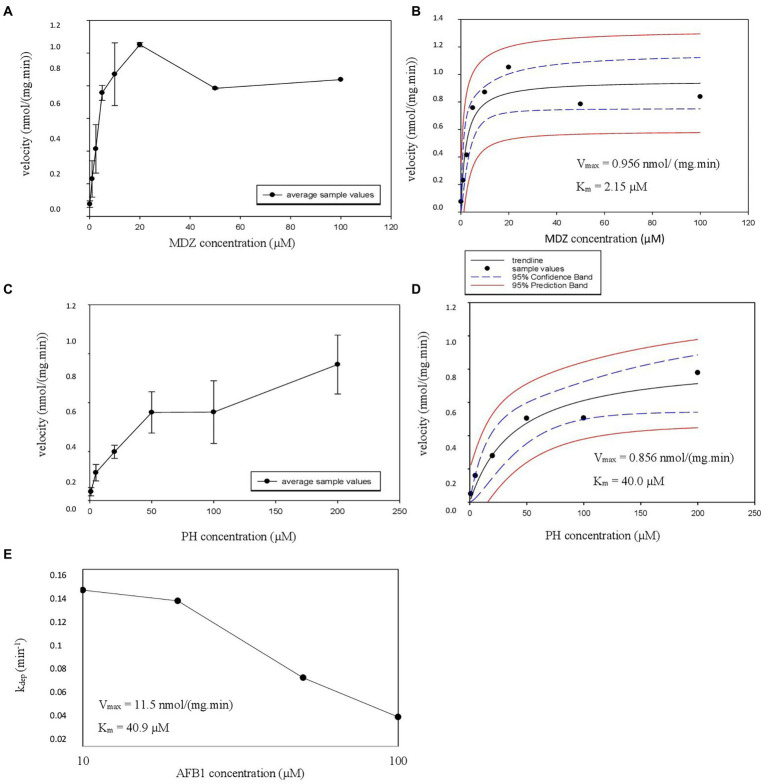
**(A–E)** Michaelis Menten constant (*K_m_*), estimated maximum velocity (*V*_max_) HLM experiment graphs. **(A,C)** The velocity as a function of the added CYP probe concentration is illustrated with error bars. The formation of hydroxy-midazolam (OH-MDZ) and acetaminophen (AC) is given from top to bottom. **(B,D)** The nonlinear regression Michaelis–Menten curves are given with the estimated *V*_max_ and *K_m_* values, also from top to bottom in the same order, 95% confidence band and 95% prediction band are presented as well. **(E)** Represents the parent depletion of AFB1 where k_dep_ is represented on the y-axis and aflatoxin B1 (AFB1) concentration on the x-axis with the estimated *V*_max_ and *K_m_* values.

Using the results of the linearity experiments, *K_m_* and *V*_max_ experiments were performed in HLM. First, metabolite formation velocity *versus* substrate concentration Figures were plotted, followed by nonlinear regression to estimate the *V*_max_ and associated *K_m_* values (Figures 2A–D). For AFB1, where substrate depletion was analyzed, *k*_dep_ was determined for different substrate concentrations and was plotted against AFB1 substrate concentrations on a log-scale (Figure 2E; Youdim and Dodia, 2010). The *K_m_* and *V*_max_ values obtained in HLM for AFB1 are summarized in Table 3.

**Table 3:** Overview of the *K_m_*, *V*_max_, ISEF corrected *V*_max_, CL_int,*in vitro*_, ISEF corrected CL_int,*in vitro*_, CL_H,u,int_, AAF corrected CL_H,u,int_ and CL_H,B_ for all experiments concerning AFB1 both in HLM and in rhCYPs.

*In vitro* system	*K_m_*	*V*_max_	*V*_max,ISEF_	CL_int,*in vitro*_	CL_int,*in vitro*,ISEF_	CL_H,u,int_		CL_H,u,int,AAF_	CL_H,B_
	(μM)	[nmol/(mg.min)]		(μl/mg.min)		(L/h)			
HLM	40.9	11.5	–	282	–	1,190	535^CYP3A4^	450^CYP3A4^	40.0^CYP3A4^
							583^CYP1A2^	420^CYP1A2^	38.5^CYP1A2^
Supersomes (CYP3A4)	49.6	12.1	5.97	243	179	755		634	47.7
EasyCYP bactosomes (CYP1A2)	58.2	10.4	14.7	178	308	1,295		932	56.1

AAF, activity adjustment factor; AFB1, aflatoxin B1; CL_H,B_, hepatic blood clearance; CL_H,u,int_, unbound intrinsic hepatic clearance; CL_int,*in vitro*_, *in vitro* determined intrinsic clearance; CYP, cytochrome P; HLM, human liver microsomes; ISEF, intersystem extrapolation factor; *K_m_*, Michaelis–Menten constant; rhCYPs, recombinant enzyme systems; *V*_max_, maximal reaction velocity.

The CL_int,*in vitro,*HLM_ (Equation 1) for CYP3A4 (midazolam 1′ hydroxylation), CYP1A2 (phenacetin O-dealkylation) and AFB1 (overall metabolism) were 445 μl/(min.mg), 21.4 μl/(min.mg), and 282 μl/(min.mg), respectively. An MPPGL and LW of 40 mg/g and 1,650 g (Equation 2) were used (Table 4) to calculate a hepatic intrinsic clearance as described in literature allowing direct comparison between literature results and the obtained experimental results. The obtained CL_H,u,int_ (Equation 4) were 1,941 L/h (MDZ), 84.70 L/h (PH), and 1,190 L/h (AFB1). The CL_H,u,int_ values need to be extrapolated to the whole liver hepatic clearance *in vivo*, taking into account the AAF (Equation 6), free fraction in blood (f_u,B_), the hepatic blood flow (Q_H_) and in case of rhCYP systems, the ISEF (Equation 8). The formulas used for the extrapolation to the whole liver hepatic clearance are shown in Equation 9 for HLM experiments and in Equation 10 for rhCYP experiments. For AFB1, an f_u,B_ of 0.16 was reported by Gilbert-Sandoval et al., 2020; the f_u,mic_ (0.9399) was estimated with Equation 3 for neutral compounds using SimCYP. The resulting CL_H,B_ are listed in Table 5.

**Table 4:** Overview of intrinsic *in vitro* clearance and intrinsic *in vivo* clearance for certain CYP probes and AFB1.

Substrate	Reaction	CL_int,*in vitro*_ [μl/(min.mg)]	CL_H,u,int_ (L/h) (whole liver)		Number of donors in HLM pool	Reference
Midazolam CYP3A4	Midazolam 1′ hydroxylation	445	1,941			150	Experimental
		464	Not mentioned			105	(Gao et al., 2017)
		693	2,700			16.0	(Kazmi, 2015)
Phenacetin CYP1A2	Phenacetin O-dealkylation	21.4	84.74			150	Experimental
		14.5	Not mentioned			105	(Gao et al., 2017)
		23.3	92.00			16.0	(Kazmi, 2015)
Aflatoxin B1	Overall metabolism	282	1,190	535^*^^CYP3A4^	150	Experimental
				583^*^^CYP1A2^		

* CL_H,u,int_ for CYP3A4 and CYP1A2 based on the contribution determined via the inhibition assay.

CL_H,u,int_, unbound intrinsic hepatic clearance; CL_int,*in vitro*_, *in vitro* determined intrinsic clearance; CYP, cytochrome P; HLM, human liver microsomes.

**Table 5:** Overview of the calculated hepatic clearance and the *in vivo* hepatic clearance retrieved from literature.

Substrate	f_u,B_	f_u,mic_	B:P	CL_H,B_ calculated	CL_H,__B_ literature	CL_H,p_ calculated	CL_H,p_ literature
MDZ	0.0531^a^	0.907^a^	0.603^a^	44.1 L/h	44.4 L/h^c^	26.6 L/h	29.0 L/h^d^
PH	0.469^a^	1^a^	1.01^a^	21.7 L/h	20.8 L/h^e^ 19.9–474.9 L/h^f^	21.9 L/h	24.1 L/h^e^
AFB1 (CYP3A4)^HLM^	0.160^b^	0.940^a^	1.03^a^	40.0 L/h		41.2 L/h	
AFB1 (CYP1A2)^HLM^	0.160^b^	0.940^a^	1.03^a^	38.5 lL/h		39.6 L/h	
AFB1 (CYP3A4)^rhCYP^	0.160^b^	0.940^a^	1.03^a^	47.7 L/h		49.1 L/h	
AFB1 (CYP1A2)^rhCYP^	0.160^b^	0.940^a^	1.03^a^	56.1 L/h		57.8 L/h	

a SimCYP.

b Gilbert-Sandoval et al. (2020).

c Kazmi (2015).

d Persson et al. (1987).

e Shibata et al. (2002).

f Kellermann and Luyten-Kellermann (1978).

AFB1, aflatoxin B1; B:P, blood to plasma ratio; CL_H,B_, hepatic blood clearance; CL_H_,_p_, hepatic plasma clearance; CYP, cytochrome P; f_u,B_, free fraction in blood; f_u,mic_, free microsomal fraction; HLM, human liver microsomes; MDZ, midazolam; PH, phenacetin; rhCYP, recombinant enzyme system.

**Figure 3:**
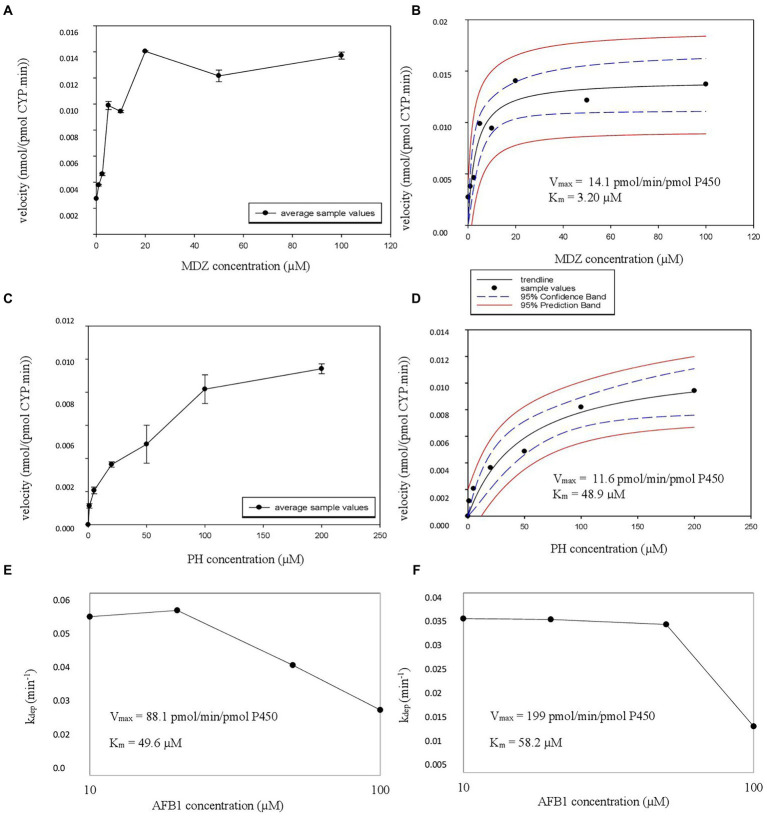
**(A–E)** Michaelis Menten constant (*K_m_*), estimated maximum velocity (*V*_max_) experiment rhCYP CYP3A4 graphs. **(A,C)** The velocity in nmol/(pmol CYP.min) in function of the added substrate concentration in μM is illustrated with error bars. The formation of alpha-hydroxy-midazolam (α-OH-MDZ) is given at the top, formation of aflatoxin B1 (AFB1) CYP3A4 metabolites is given at the bottom. **(B,D)** The nonlinear regression Michaelis–Menten curves are given with the *V*_max_ and *K_m_* values, top for midazolam (MDZ) and bottom for AFB1. **(E,F)** The substrate depletion is represented by the depletion rate (*k*_dep_) and is plotted against AFB1 concentration in μM for CYP3A4 supersomes **(E)** and for CYP1A2 bactosomes **(F)**.

Next, *K_m_* and *V*_max_ experiments were performed in recombinant enzyme systems. CYP3A4 supersomes were employed for MDZ and AFB1 and EasyCYP LR CYP1A2 bactosomes for PH and AFB1. Metabolite formation velocity *versus* substrate concentration Figures were plotted, again followed by nonlinear regression to estimate the *V*_max_ and associated *K_m_* values for MDZ (Figures 3A,B) and PH (Figures 3C,D). For AFB1, where substrate depletion was analyzed, k_dep_ was determined for different substrate concentrations in supersomes and bactosomes which was plotted against AFB1 substrate concentrations on a log-scale (Figures 3E,F). By dividing the theoretical maximal k_dep_ by the protein concentration, the CL_int_ was achieved from which the *V*_max_ could be determined (Youdim and Dodia, 2010). The *K_m_* and *V*_max_ values obtained in rhCYPs for AFB1 are summarized in Table 3.

The obtained rhCYP *K_m_* and *V*_max_ values of the CYP3A4 probe MDZ, were used to calculate the ISEF for both *V*_max_ and CL_int_ (Equations 7 and 8). The CYP3A4 abundance of 137 pmol/mg protein was used as provided by SimCYP. A *V*_max_ ISEF of 0.495 and a CL_int_ ISEF of 0.737 were obtained. When using the data of AFB1 obtained in rhCYP experiments for extrapolation to HLM values, using the determined ISEFs, a *V*_max,ISEF_ of 43.6 pmol/min/pmol P450 and a CL_int,ISEF_ of 179 μl/(mg min) were obtained. Next, the AAF was calculated using the HLM values of the probe substrate MDZ for CYP3A4 and PH for CYP1A2. The *in vivo* CL_int_ was determined with the information obtained from Gertz et al. (2010), where the total hepatic clearance of MDZ was given. With the use of SimCYP, it was calculated that 88% of the MDZ metabolism is performed by CYP3A4. For CYP1A2, the information obtained from Koganti et al. (2005) was used, where SimCYP predicted a 74% contribution, which was applied to the overall *in vivo* CL_int_ from literature. Furthermore, an MPPGL of 40 mg/g and a LW of 1,650 g were taken into account for the determination of CL_int,u,*in vivo*,CYP_. The f_u,mic_ for MDZ is 0.83 with a microsomal protein concentration of 0.50 mg/ml. With the use of Equation 5, an f_u,mic_ of 0.907 was achieved for a microsomal protein concentration of 0.25 mg/ml. An AAF of 0.84 was obtained with Equation 6, implying that the *in vivo* activity of CYP3A4 is 1.19-fold lower than the *in vitro* CYP3A4 activity. Using Equation 10 on the CL_H,int,u_ to calculate the CL_H,B_ of AFB1 for CYP3A4, as determined in supersomes, a value of 47.7 L/h was acquired which is 1.19 times the CL_H,B_ of AFB1 for CYP3A4 of 40.0 L/h determined in HLM (Table 3). Applying the CYP3A4 AAF to the experimentally determined CL_H,int,u_, a CL_H,B_ for MDZ of 44.1 L/h was achieved which is close to the *in vivo* human hepatic blood clearance of 44.4 L/h reported by Kazmi (2015; Table 5). The obtained rhCYP *K_m_* and *V*_max_ values of the CYP probe PH were used to calculate the ISEF for both *V*_max_ and CL_int_ (Equation 7 and 8). The CYP1A2 abundance of 52 pmol/mg protein was used as provided by SimCYP. A *V*_max_ ISEF of 1.42 and a CL_int_ ISEF of 1.73 were obtained. A *V*_max,ISEF_ of 14.7 pmol/min/pmol P450 and a CL_int,ISEF_ of 308 μl/(mg.min) were obtained for AFB1 using the rhCYP experimental data. For CYP1A2, an AAF of 0.72 was calculated, implying that the *in vivo* activity of CYP1A2 is 1.39-fold lower than the *in vitro* CYP1A2 activity. When using the AAF for CYP1A2 to the obtained values of AFB1 in HLM and in rhCYPs, hepatic blood clearances of 38.5 L/h and 56.1 L/h were obtained, respectively (Table 3). Applying the CYP1A2 AAF to the experimentally determined CL_H,int,u_, a CL_H,B_ for PH of 21.7 L/h was achieved which is close to the *in vivo* human hepatic blood clearance of 20.8 L/h reported by Shibata et al. (2002) and lies within the range reported by Kellermann and Luyten-Kellermann (1978; Table 5).

### AFB1 inhibition assays

For the inhibition assays in HLM with AFB1, CYP3A4/3A5 enzymes were inhibited by ketoconazole and CYP1A2 by α-naphthoflavone. A first-order rate constant, incubation volume, amount of microsomal protein, an MPPGL of 40 mg/g and a LW of 1,650 g were used to determine the CL_int,u_. Based on the ratio of the difference in CL_int,u_, both without and with inhibitor, and the CL_int,u_ without inhibitor, a CYP3A4/5 contribution of 45% and a CYP1A2 contribution of 49% were obtained. Both contributions sum up to a total of 94%.

## Discussion

### Optimal incubation conditions of CYP probes and AFB1 and AFB1 inhibition assays

For AFB1, additional timepoints were chosen at the beginning of the reaction since ideal incubation times are often below 20 min (Di et al., 2004). In addition, only protein levels of 0.25 mg/ml and 0.5 mg/ml were tested for AFB1 linearity determination. It was not feasible to monitor all AFB1 metabolites since not all reference standards were commercially available for the development of a LC–MS/MS method. Therefore, we opted to monitor the depletion of AFB1, instead. However, this approach is less accurate than following metabolite formation. The reduction of the parent compound is quantified by analyzing the parent compound before and after incubation. Only a fraction of the parent compound is metabolized, therefore, the difference in the absolute peak areas of the parent compound will be less accurate than directly monitoring the metabolites. Since two peak areas are quantified (before and after) instead of one (metabolite) and only a small part is metabolized, the error on the disappearance of the parent compound will be higher. Nevertheless, this is the current *go-to* method if not all metabolites are available as analytical standards. Since parent compound depletion was monitored in case of AFB1, the *K_m_* was determined using the depletion rate constants as described by Youdim and Dodia (2010). The ideal microsomal protein concentration and incubation times for *K_m_* and *V*_max_ determination were determined for MDZ, PH and AFB1. From these experiments, it can be concluded that incubation times are often below 20 min and microsomal protein concentrations are approximately 0.25–0.5 mg/ml to maintain linear conditions.

When choosing optimal conditions (microsomal protein concentration and incubation time), two criteria must be fulfilled, i.e., not more than 20% of the initial amount of substrate compound can be metabolized, assuring the maintenance of initial rate conditions, and saturation may not be reached, since optimal conditions require that experiments are performed in the linear range (with enzyme concentration and time; Ruikar and Rajput, 2012; Wang et al., 2014).

Inhibition assays showed that CYP3A4/3A5 is involved in 45% of the overall AFB1 hepatic metabolism, 49% is attributed to CYP1A2 and the other 6.0% can be ascribed to other enzymes, expectedly CYP2A13 (He et al., 2006). These results agree with Gallagher et al. (1996), where CYP1A2 was indicated as main metabolizing CYP450 enzyme of AFB1. Gallagher et al. (1996) stated a 95% contribution of CYP1A2 in case of low exposure (0.133 μM) to AFB1, supporting that CYP1A2 is the predominant enzyme in AFB1 metabolism. Data from experiments with higher AFB1 levels (25–500 μM) report a CYP1A2 contribution of only 1–5% whilst CYP3A4 is attributed to 79–95% and CYP3A5 and CYP3A7 to 4–15% and 5–7%, respectively (Kamdem et al., 2006). The difference in CYP1A2 and CYP3A4 contribution for different AFB1 exposures could be assigned to different CYP450 isoenzyme kinetics. Kamdem et al. (2006) stated that CYP3A4 bioactivation follows Hill kinetics whereas CYP1A2 bioactivation follows Michaelis–Menten kinetics. Hill kinetics follow a deviation on the hyperbolic shape of the Michaelis–Menten kinetics (where the coefficient n equals 1). In case of Hill kinetics, a positive or negative kinetic cooperativity is observed were *n* > 1 or *n* < 1, respectively (Emelyanova, 2018). In general, one can state that *in vivo* AFB1 exposure is considered as low-dose AFB1 intake (World Health Organization, 2018) and will be metabolized predominantly by CYP1A2, following Michaelis–Menten kinetics. In case of higher *in vivo* exposure, CYP3A4 has the predominant role, following Hill kinetics. It is important to have insight in the involved enzymes in the metabolism of a compound in order to understand what happens in the human body. Furthermore, it is crucial to predict possible interactions with other substances that are metabolized by the same enzymes or that have an inducing or inhibiting effect on the concerning enzymes. Interaction at the level of CYP450 enzymes might lead to a higher toxicity or less effectiveness, in case of therapeutic drug substances.

### *K_*m*_ V*_max_ determination of CYP probes and AFB1

Pharmacokinetic parameters were performed on CYP probe substrates to verify the validity of the used experimental design and to determine ISEFs and AAFs. For MDZ, a *K_m_* of 2.15 μM was found with a *V*_max_ of 956 pmol/(mg.min), which is acceptable considering earlier reported values for MDZ [*K_m_* range: 1.9–9.0 μM; *V*_max_ range: 190.0–4,380 pmol/(mg.min); Von Moltke et al., 1996; Hickman et al., 1998; Yuan et al., 2002; Bian et al., 2015; Kazmi, 2015; Nguyen et al., 2016; Gao et al., 2017]. For PH, a *K_m_* of 40.0 μM was observed with a *V*_max_ of 856 pmol/(mg.min), which is consistent with earlier reported values for PH [*K_m_* range: 10–62 μM; *V*_max_ range: 241–2,173 pmol/(mg.min); Boobis et al., 1981; Gillam and Reilly, 1988; Yuan et al., 2002; Gao et al., 2014, 2017; Erickson et al., 2015; Kazmi, 2015].

For the *K_m_* and *V*_max_ determination of AFB1, two approaches were applied: one with the use of HLM, where an overall *K_m_* and *V*_max_ were determined, and another one where rhCYPs were used to determine CYP-specific *K_m_* and *V*_max_, with CYP3A4 and CYP1A2 as main metabolizing enzymes for AFB1 (He et al., 2006; Kamdem et al., 2006; Bbosa et al., 2013). For AFB1, the overall *K_m_* and *V*_max_ in HLM are 40.9 μM and 11,536 pmol/(mg.min). A paper by Gallagher et al. (1994) reported *K_m_* values of 41 μM for the AFBO formation via CYP1A2, 29 μM for the AFM1 formation via CYP1A2, 133 μM for the AFBO formation via CYP3A4 and 139 μM for the AFM1 formation via CYP3A4. The liver microsomes from 3 male subjects (age range 21–46 years old) were used in these experiments (Gallagher et al., 1994). Data from Kamdem et al. (2006) reported *K_m_* values from 13 HLM donors following AFBO or AFQ1 formation ranging from 90–1,720 μM with associated *V*_max_ values ranging from 236–11,281 pmol/(mg.min). The performed supersome experiments resulted in a *K_m_* value of 49.6 μM and a *V*_max_ value of 88.1 pmol/min/pmol P450 for CYP3A4 metabolism; the performed CYP1A2 bactosome experiments resulted in a *K_m_* value of 58.2 μM and a *V*_max_ value of 199 pmol/min/pmol P450. Since limited information has thus far been presented on the PK parameters of mycotoxins, especially not on the overall *K_m_* and *V*_max_ of AFB1, more in-depth comparison with literature data proved impossible. Reported *K_m_* and *V*_max_ determinations cover a large range of values and are dependent on a variety of parameters such as the applied HLM and type of HLM donors (ethnicity, pathologies etc.). Furthermore, it is important to take into account the HLM pool size and to perform experiments on probe substrates, so environmental laboratory parameters do not have an influence. The determined *V*_max_ from the current experiments lies within the ranges reported earlier for AFB1 metabolization. The determined *K_m_* cannot be compared since an overall *K_m_* via HLM, a *K_m_* based on CYP3A4 metabolism and a *K_m_* based on CYP1A2 metabolism using rhCYPs were determined. Firstly, the earlier reported *K_m_* values are for a specific metabolite formation whereas this study looked at the overall metabolism via HLM or the metabolism by a specific CYP450 enzyme. CYP1A2 forms both AFBO and AFM1, whereas CYP3A4 is involved in AFBO and AFQ1 formation. Secondly, the current experiment used an HLM pool of 150 donors consisting of both male and female subjects whereas, in Gallagher et al. (1994) the number of microsome donors was only 3, and in the Kamdem et al. (2006) it was only 13. Thirdly, Kamdem et al. (2006) reported a substantial interindividual difference in enzyme expression, which had an enormous impact on AFB1 activation to AFBO and deactivation into other metabolites (lower or higher metabolism). Therefore, it is very important to pool large amounts of donors to avoid major impact of interindividual variability. The *K_m_* values from Kamdem et al. (2006) and Gallagher et al. (1994) do not really correspond, also not with the currently reported *K_m_* data. The former published data lack a representative HLM pool, therefore it can be stated that the results of this paper show a representative *K_m_* and *V*_max_ value for AFB1 in HLM. This info can be used for further co-incubation experiments where a concentration below the *K_m_* value (preferably 1/10th of the *K_m_* so that the substrate concentration does not have an impact on the CL_int,*in vitro*_) is needed. Based on the experimental *K_m_* values, the respective concentrations would likely be set at 0.2 μM, 4 μM and 5 μM for MDZ, PH and AFB1, respectively.

The calculated CL_int,*in vitro*_ for CYP3A4 (midazolam 1′ hydroxylation), CYP1A2 (phenacetin O-dealkylation) and the overall metabolism of AFB1 from HLM experiments are 445 μl/(mg.min), 21.4 μl/(mg.min) and 282 μl/(mg.min), respectively. Using f_u,B_ and f_u,mic_ values found in literature, an MPPGL of 40 mg/g, a LW of 1,650 g and experimentally determined AAFs and CYP-contributions, a CL_H,B_ of 44.1 L/h (MDZ), 21.7 L/h (PH), 40.0 L/h (AFB1 CYP3A4) and 38.5 L/h (AFB1 CYP1A2) were obtained. Compared to literature, these values were within 1.00 and 1.04-fold for MDZ (Kazmi, 2015) and PH (Shibata et al., 2002).

The CL_int, *in vitro*_ values from rhCYP experiments on AFB1 were used for the extrapolation to *in vivo* CL_H,B_. A *V*_max_ ISEF of 0.495 and a CL_int_ ISEF of 0.737 were obtained for CYP3A4 supersomes. The correlated recombinant system *K_m_* was 49.6 μM and *V*_max_ was 88.1 pmol/min/pmol P450 for AFB1, while for MDZ a *K_m_* of 3.20 μM and *V*_max_ of 14.1 pmol/min/pmol P450 were obtained. After applying the determined ISEFs and AAF, the obtained CL_H,B_ for AFB1 by using rhCYPs (supersomes) was 47.7 L/h which is 1.19-fold higher than the value from HLM experiments (40.0 L/h) following the CYP3A4 pathway. A *V*_max_ ISEF of 1.42 and a CL_int_ ISEF of 1.73 were obtained from CYP1A2 bactosomes. The correlated recombinant *K_m_* was 58.2 μM and *V*_max_ was 199 pmol/min/pmol P450 for AFB1, while for PH a *K_m_* of 48.9 μM and *V*_max_ of 11.6 pmol/min/pmol P450 were obtained. After applying the determined ISEFs and AAF, the obtained CL_H,B_ for AFB1 by using rhCYPs (EasyCYP bactosomes) was 56.1 L/h which is 1.46-fold higher than the CYP1A2 value from HLM experiments (38.5 L/h). It can therefore be concluded that both HLM and rhCYPs are useful *in vitro* systems to determine PK parameters. In case of unavailability of analytical standards, CYP450-specific systems such as supersomes or bactosomes are deployed, if ISEFs are determined as well, to solve the problem of not being able to follow a specific metabolite formation. By determining the PK parameters of a known compound such as MDZ and PH, the ISEFs can be determined, necessary for extrapolation from i*n vitro* to *in vivo* and also to compensate for interindividual CYP abundance variation. These experiments showed that the supersomes (CYP3A4) had a result which was closer to the result of the HLM, compared to the easyCYP LR bactosomes (CYP1A2). This might be attributable to the use of different rhCYPs, but since a correction was made using both ISEFs and AAFs, the difference of various rhCYP systems should be negligible. Further research should be performed in order to further conclude on the performance of the different rhCYP systems.

Using the AAF, the blood CL_H,MDZ_ was determined at 44.1 L/h, which is close to the *in vivo* CL_H_ value reported in literature of 44.4 L/h (Kazmi, 2015). The hepatic *in vivo* blood clearance of AFB1 via CYP3A4 determined by HLM and rhCYP *in vitro* systems, are 40.0 L/h and 47.7 L/h, respectively, taking into account the AAF of CYP3A4 (Table 3). There is a difference between the determined HLM and rhCYP hepatic *in vivo* blood clearance. This might be attributable to the determination of the overall clearance and the application of CYP450-contributions from the inhibition assay in case of HLM clearance whereas in the case of supersomes, only the clearance due to CYP3A4 was determined. The blood CL_H,__PH_ was determined at 21.7 L/h, which is in line with the *in vivo* CL_H,B_ reported in literature of 20.8 L/h for PH (Shibata et al., 2002) and which also lies within the reported range of 19.9–474.9 L/h (Kellermann and Luyten-Kellermann, 1978). The broad *in vivo* CL_H,B_ range of PH in literature can be explained by the large interindividual variation in the first pass effect. The *in vivo* hepatic blood clearance of AFB1 via CYP1A2 determined using HLM and rhCYP, are 38.5 L/h and 56.1 L/h, respectively, taking into account the AAF of CYP1A2 (Table 3).

The implementation of AAFs results in a value closer to the *in vivo* conditions since it accounts for batch differences between *in vitro* systems, for experimental discrepancies and differences between *in vitro* and *in vivo* data. Since the implementation led to a more accurate estimation of the *in vivo* hepatic clearance of the probe substrates MDZ and PH, for CYP3A4 and CYP1A2, the AAF was applied on the CL_H,int,u_ of AFB1, for which only limited PK information is available (Jubert et al., 2009; T’jollyn et al., 2017). *In vitro* systems can be used for the determination of *K_m_*, *V*_max_ and the intrinsic clearance. Noteworthy, *in vitro* systems seem to underestimate the *in vivo* clearance (Bowman and Benet, 2019). Although this was not the case for the hepatic blood clearances of the CYP probe substrates in these experiments, it must be taken into account for the reported values of AFB1. For the extrapolation to whole liver clearance or even CL_po_, other important factors such as transporters, protein binding, bioavailability should be determined and taken into account. In conclusion, the hepatic blood clearances corrected for the AAF, i.e., CL_H,B,MDZ,AAF_, CL_H,B,PH,AAF_ CL_H,B,AFB1,AAF(CYP3A4)_ and CL_H,B,AFB1,AAF(CYP1A2)_ were determined in HLM at 44.1 L/h, 21.7 L/h, 40.0 L/h and 38.5 L/h. Inhibition assays in HLM showed that 45% of the AFB1 metabolism was performed by CYP3A4/3A5 enzymes and 49% by CYP1A2 enzymes. In HLM the overall *K_m_* was 40.9 μM and the *V*_max_ was 11.5 nmol/(mg.min) for AFB1. In recombinant enzyme systems, the *K_m_* and *V*_max,AAF_ were 49.6 μM and 43.6 pmol/min/pmol P450 for CYP3A4 and 58.2 μM and 283 pmol/min/pmol P450 for CYP1A2.

## Data availability statement

The original contributions presented in the study are included in the article/supplementary material, further inquiries can be directed to the corresponding authors.

## Author contributions

OL: conceptualization, writing original draft, and correcting draft. MB, EG, and AV: conceptualization, supervision and review original draft, and final approval. JB and SS: supervision and review original draft, and final approval. All authors contributed to the article and approved the submitted version.

## Conflict of interest

The authors declare that the research was conducted in the absence of any commercial or financial relationships that could be construed as a potential conflict of interest.

## Publisher’s note

All claims expressed in this article are solely those of the authors and do not necessarily represent those of their affiliated organizations, or those of the publisher, the editors and the reviewers. Any product that may be evaluated in this article, or claim that may be made by its manufacturer, is not guaranteed or endorsed by the publisher.
